# Formula feeding and immature gut microcirculation promote intestinal hypoxia, leading to necrotizing enterocolitis

**DOI:** 10.1242/dmm.040998

**Published:** 2019-12-09

**Authors:** Yong Chen, Yuhki Koike, Lijun Chi, Abdalla Ahmed, Hiromu Miyake, Bo Li, Carol Lee, Paul Delgado-Olguín, Agostino Pierro

**Affiliations:** 1 Translational Medicine, The Hospital for Sick Children, Toronto, ON M5G0A4, Canada; 2 Division of General and Thoracic Surgery, The Hospital for Sick Children, Toronto, ON M5G1X8, Canada; 3 Department of Pediatric Surgery, KK Women's and Children's Hospital, 100 Bukit Timah Road, 229899 Singapore; 4 Department of Molecular Genetics, University of Toronto, Toronto, ON M5S1A8, Canada; 5 Heart and Stroke Richard Lewar Centre of Excellence in Cardiovascular Research, Toronto, ON M5S3H2, Canada

**Keywords:** Necrotizing enterocolitis, Microvascular flow dynamics, Vascular maturation, Premature intestine circulation, Microvasculature dilation, Hypoxia

## Abstract

Major risk factors for necrotizing enterocolitis (NEC) are formula feeding and prematurity; however, their pathogenic mechanisms are unknown. Here, we found that insufficient arginine/nitric oxide synthesis limits blood flow in the intestinal microvasculature, leading to hypoxia, mucosal damage and NEC in the premature intestine after formula feeding. Formula feeding led to increased intestinal hypoxia in pups at postnatal day (P)1 and P5, but not in more mature pups at P9. Accordingly, blood flow in the intestinal microvasculature increased after formula feeding in P9 pups only. mRNA profiling revealed that regulators of arginine/nitric oxide synthesis are at higher levels in endothelial cells of the intestine in P9 than in P1 pups. Importantly, arginine supplementation increased intestinal microvasculature blood flow and prevented NEC, whereas an arginine antagonist exacerbated NEC. Our results suggest that balancing intestinal oxygen demand and supply in the premature intestine by modulating arginine/nitric oxide could be used to prevent NEC.

This article has an associated First Person interview with the first author of the paper.

## Abstract (precis)

**Summary:** We demonstrate that the immature gut is prone to necrotizing enterocolitis due to limited arginine/nitric oxide synthesis, leading to inadequate microcirculation in response to the challenge imposed by formula feeding.

## INTRODUCTION

Necrotizing enterocolitis (NEC) is the most common and devastating gastrointestinal emergency in premature neonates (Holman et al., 2006; Neu and Walker, 2011; Niño et al., 2016). During the past few decades, the overall incidence of NEC has not decreased and the mortality rate remains high (15-30%) (Chen et al., 2016a; Fitzgibbons et al., 2009; Hein-Nielsen et al., 2015; Holman et al., 2006).

Prematurity and enteral formula feeding are two of the most important risk factors for NEC, but their contribution to NEC pathogenesis is unclear (Kosloske, 1994; Llanos et al., 2002; Neu and Walker, 2011). More than 90% of infants with NEC have been enterally fed (Kosloske, 1994; Llanos et al., 2002). Premature infants tend to develop NEC after 2-4 weeks of being parenterally fed after birth (Guthrie et al., 2003; Stout et al., 2008). We demonstrated that formula feeding is strongly associated with intestinal ischaemia and hypoxia in human NEC, but the mechanism is not clear (Chen et al., 2016b).

Digestion and absorption are high-energy-consuming processes. Absorption depends on active transport, which consumes adenosine triphosphate and O_2_ (Nowicki et al., 1983; Ward et al., 2014). To meet the increased O_2_ demand after feeding, intestinal blood flow increases by 200% above the baseline in adults, a process known as postprandial hyperaemia (Sieber et al., 1991). Intestine blood flow is primarily modulated by the endothelium-produced vasodilator nitric oxide (NO) and the vasoconstrictor endothelin-1 (ET-1) (Watkins and Besner, 2013). NO is mainly produced from its precursor, arginine, by endothelial NO synthase (eNOS). Deletion of the eNOS-encoding gene *Nos3* results in early-onset and exacerbated NEC (Yazji et al., 2013), and a variant of carbamoyl phosphate synthase 1 (CPS1), a rate-limiting enzyme in arginine biosynthesis, is associated with NEC susceptibility in preterm infants (Moonen et al., 2016).

Prematurity is strongly associated with NEC, with its highest incidence in infants with extremely low birth weight (ELBW) (Kosloske, 1994). The premature gut expresses high levels of Toll-like receptor 4 (TLR4), which binds bacterial lipopolysaccharide (LPS) to activate innate immunity (Niño et al., 2016). TLR4 promotes NEC by inducing inflammation, inhibiting enterocyte proliferation and reducing intestinal microcirculation (Egan et al., 2016; Pearson et al., 2013; Yazji et al., 2013). Deletion of TLR4 in endothelial cells increases eNOS/NO-dependent intestinal microcirculation and reduces the incidence of NEC (Yazji et al., 2013). NO production is also limited by the amount of its precursor arginine, which is remarkably low in premature infants' serum (Contreras et al., 2017). Accordingly, postprandial hyperaemia is decreased in ≤2-day-old premature piglet gut compared with 2-week-old piglet gut (Yao et al., 1986). In adult humans, the mean mesentery blood velocity increases by more than 150% after enteral feeding, whereas it raises only by ∼30% in ELBW infants (Havranek et al., 2015; Sieber et al., 1991). Interestingly, postprandial hyperaemia is almost completely abolished in premature infants with feeding intolerance or large patent ductus arteriosus, which predisposes to NEC (Fang et al., 2001; Havranek et al., 2015).

Based on these findings, we hypothesized that the premature gut has a limited capacity to regulate blood flow and is thus more susceptible to ischaemic damage after aggressive feeding. In this study, we investigated how the response of the intestinal microcirculation to formula feeding is controlled, and its contribution to NEC.

## RESULTS

### Hyperosmolar formula feeding induces mucosal hypoxia in experimental NEC

**Fig. 1.**
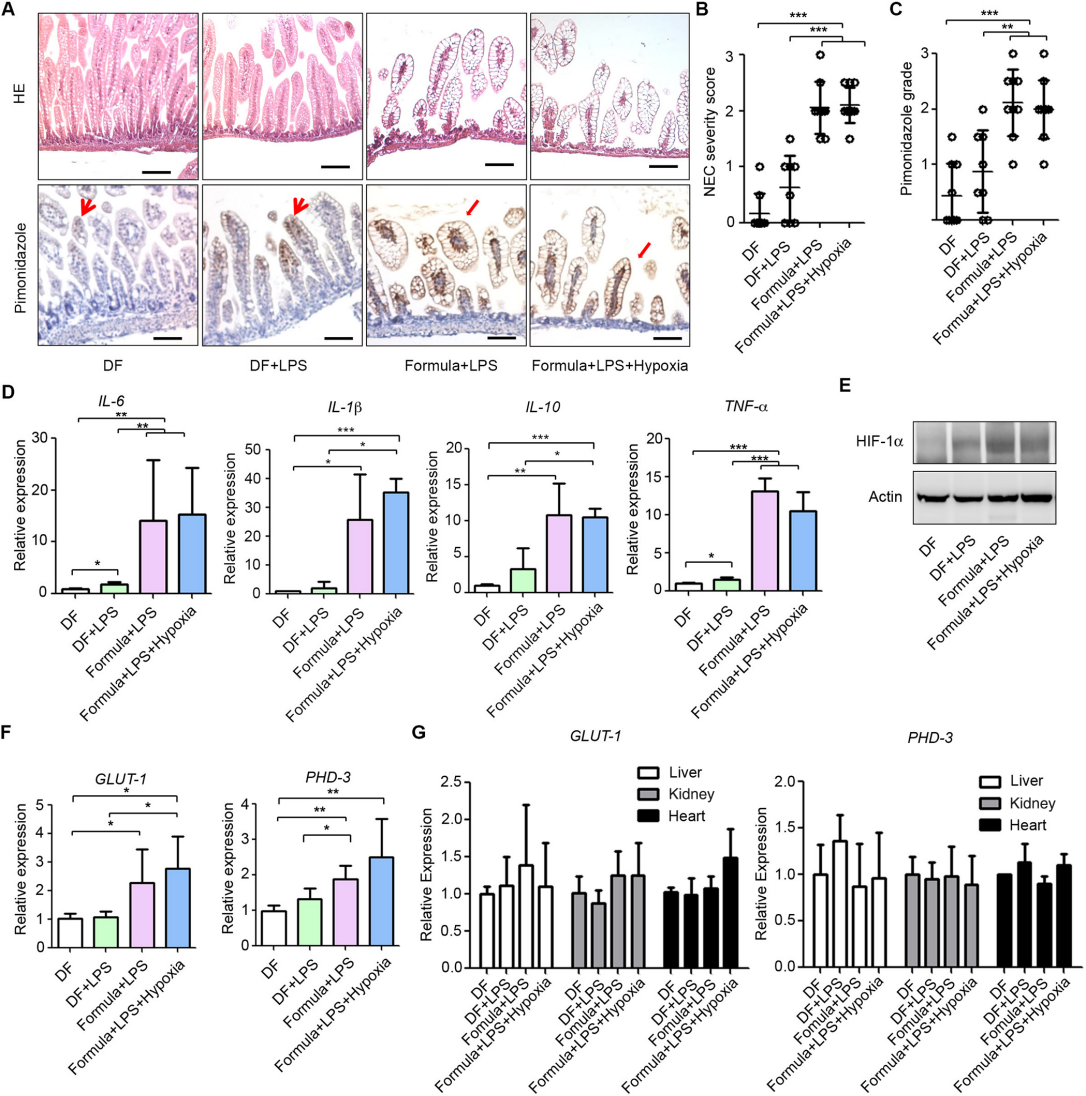
**Formula feeding induces mucosal hypoxia in experimental NEC.** (A) Haematoxylin and Eosin (HE) and pimonidazole staining in ileum from pups: dam fed (DF; *n*=9), DF+lipopolysaccharide (LPS) (*n*=8), formula+LPS (*n*=9), and formula+LPS+systemic preprandial hypoxia (*n*=10). Arrowheads point to tips of villi in DF and DF+LPS pups; arrows point to damaged villi in formula+LPS and formula+LPS+hypoxia pups. Samples were collected 90 min after feeding. Scale bars: 100 µm. (B) NEC severity score. (C) Pimonidazole grading. (D) qRT-PCR showing normalized expression levels of *Il-6*, *Il-1b*, *Il-10* and *Tnf-a* mRNA in ileum. (E) Western blot analysis of HIF-1α in ileum. (F) qRT-PCR of *GLUT-1* and *PHD-3* in ileum. (G) qRT-PCR of *GLUT-1* and *PHD-3* in liver, kidney and heart. *P<0.05, **P<0.01, ***P<0.001. All values are mean±s.d.

Hyperosmolar formula feeding exerts excessive digestion stress on the immature gut and is commonly used, together with LPS and systemic hypoxia, for inducing NEC in animals (Zani et al., 2016). We exposed P5 mouse pups to different combinations of these stressors to determine the contribution of formula feeding to intestinal hypoxia and NEC. As expected, breastfeeding of control pups (dam fed, DF) did not cause intestinal damage or induce increased levels of the marker of hypoxia pimonidazole (Fig. 1A-C). Feeding LPS to DF pups (DF+LPS) triggered a subtle increase in the inflammatory cytokine *Il-6* (Fig. 1D), but failed to induce significant intestinal injury, hypoxia or NEC (Fig. 1A-C), suggesting that inducing an inflammatory response alone is not sufficient to induce NEC. In contrast, mice fed with formula+LPS had significant mucosal damage with epithelial oedema, villous sloughing and core separation, as well as a higher NEC severity score than DF and DF+LPS pups (Fig. 1A-C). The mRNA expression of the inflammatory cytokines *Il-6*, *Il-1b*, *Il-10* and *Tnf-a* was also increased in the formula-fed groups (Fig. 1D).

By assessing the levels of pimonidazole, we detected weak staining at the tips of villi in healthy submucosa in DF pups (Fig. 1A, arrowheads). In contrast, pimonidazole staining was remarkably stronger and extended to the bottom of the villi in formula-fed groups (Fig. 1A). Similarly, the level of hypoxia-inducible factor 1α (HIF-1α), a key mediator of the response to hypoxia (Wang et al., 1995), was higher in the intestine in formula-fed pups than in DF pups (Fig. 1E). Expression of the HIF-1α targets glucose transporter 1 (*GLUT-1*; also known as *Slc2a1*) and prolyl 4-hydroxylase 3 (*PHD-3*; also known as *Egln3*) (Fujita et al., 2012; Rademakers et al., 2011) was also increased in the formula-fed pups (Fig. 1F). Expression levels of *GLUT-1* and *PHD-3* were comparable in the liver, kidney and heart in formula-fed and DF groups, indicating that intestinal hypoxia is tissue specific (Fig. 1G). In addition to formula and LPS feeding, some pups were subjected to 10 min of systemic hypoxia (5% O_2_) before each formula feed (preprandrial). Interestingly, preprandrial systemic hypoxia did not exacerbate intestinal hypoxia or mucosal damage over that observed in pups fed with LPS+formula (Fig. 1). These results suggest that the intestine requires less oxygen when fasting than after feeding.

### A single gavage formula feed induces intestinal mucosal hypoxia in the early life of neonatal mice

**Fig. 2.**
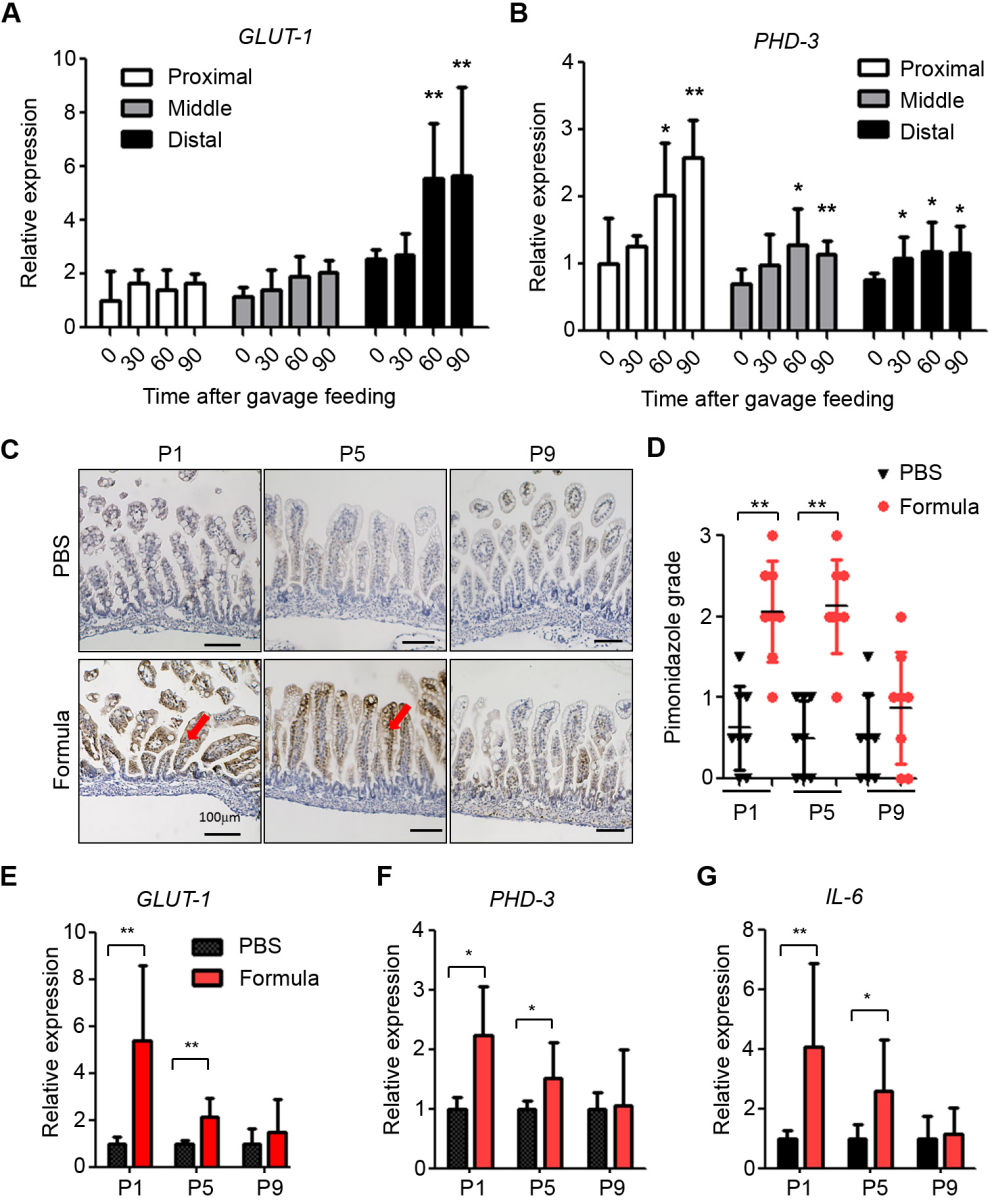
**Formula feeding induces mucosal hypoxia in experimental NEC.** (A,B) qRT-PCR of *GLUT-1* (A) and *PHD-3* (B) in proximal (jejunum), mid and distal (ileum) small intestine of P5 pups at different postfeeding time points. (C,D) Pimonidazole staining (C) and pimonidazole grading (D) of ileal tissue from P1, P5 and P9 pups fed with PBS or hyperosmolar formula (*n*=6). Scale bars: 100 µm. (E-G) qRT-PCR of *GLUT-1* (E), *PHD-3* (F) and *Il-6* (G) on ileum from P1, P5 and P9 pups fed with PBS or hyperosmolar formula. *P<0.05, **P<0.01. All values are mean±s.d.

Our findings indicate that formula feeding+LPS is required for inducing mucosal hypoxia and damage in experimental NEC (Fig. 1A). We investigated whether feeding formula only once is sufficient to trigger hypoxia in the neonatal intestine. A single feed of hyperosmolar formula in P5 pups significantly increased expression of the hypoxia marker genes *GLUT-1* and *PHD-3* in the ileum in a time-dependent manner (Fig. 2A,B). This effect is associated with osmolarity or caloric density, because feeding the same volume of iso-osmolar formula (1:1 dilution of hyperosmolar formula) induced less mucosal damage and lower pimonidazole staining than hyperosmolar formula (Fig. S1). As NEC incidence is higher in premature infants than in full-term infants (Llanos et al., 2002), we sought to determine whether feeding-induced intestinal hypoxia is associated with the age of neonatal pups. Pimonidazole staining and expression of *GLUT-1*, *PHD-3* and the inflammation inducer *Il-6* increased in the intestine of P1 and P5, but not P9, pups after a single formula feed (Fig. 2C-G). Thus, formula feeding triggers mucosal hypoxia only in the early neonatal intestine, perhaps owing to intestinal vasculature immaturity.

### Inadequate intestinal haemodynamic response to feeding in the immature intestine in early neonatal mice

**Fig. 3.**
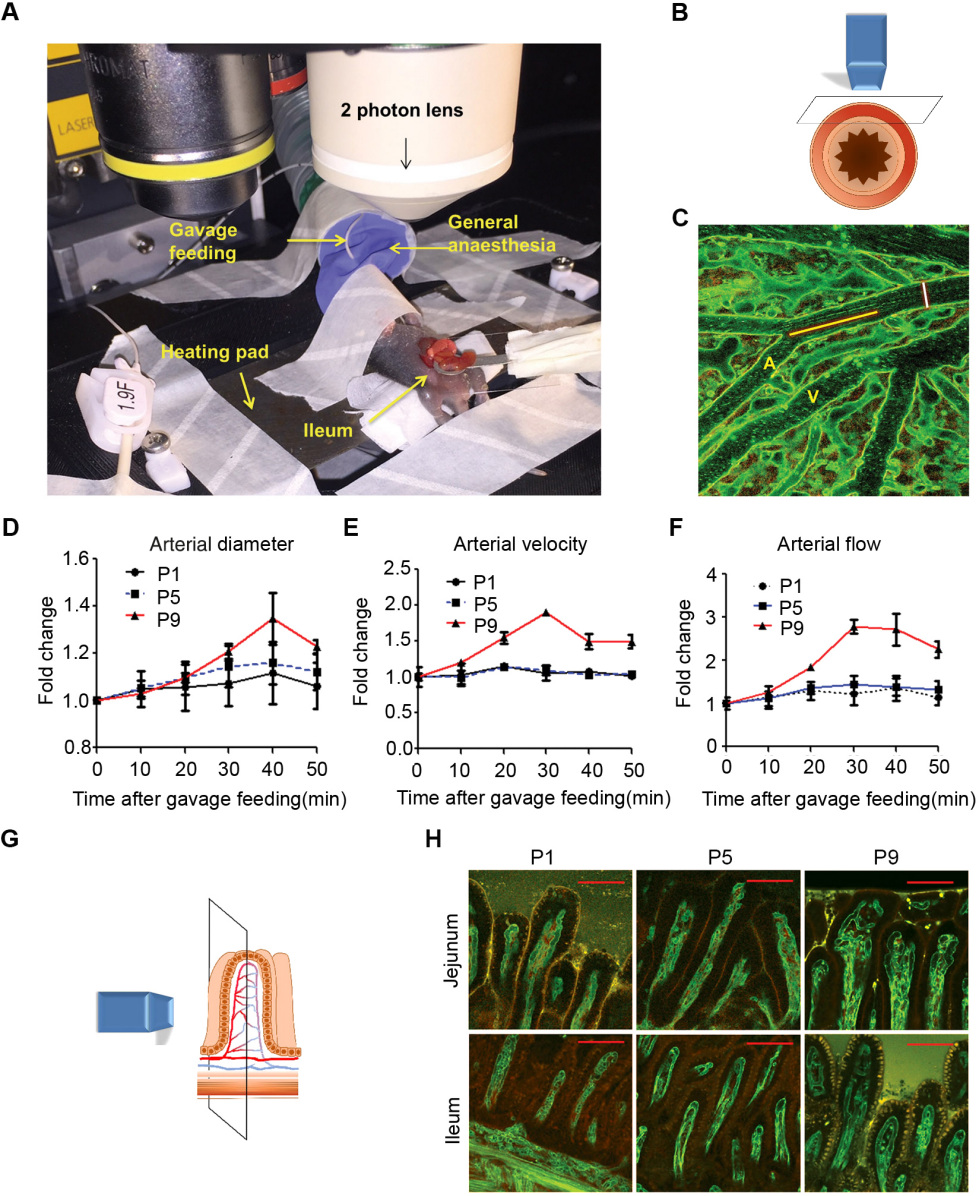
**Inadequate intestinal haemodynamic response to feeding in the immature intestine in early neonatal life.** (A) Set-up for two-photon microscopy. *Rosa^mT/mG/+^;Tie2-Cre* pups under general anaesthesia and laparotomy for visualization of the intestinal microcirculation. (B) Plane of imaging. (C) Representative image of intestinal submucosal flow using *in vivo* two-photon microscopy. A, arteriole; V, venule. White bar, arteriole diameter; yellow bar, platelet movement trajectory. (D-F) Quantification of arterial diameter (D), arterial velocity (E) and arterial flow (F) at several time points after gavage formula feeding in P1, P5 and P9 pups. (G) Plane of imaging for visualizing the villus microvasculature. (H) Representative micrographs from two-photon microscopy of the capillary network in intestinal villi of the jejunum and ileum in P1, P5 and P9 *Rosa^mT/mG/+^;Tie2-Cre* pups. At least three animals from each age group were analysed. For all graphs, error bars represent ±s.e.m. Scale bars: 100 µM.

To elucidate the basis for susceptibility of the early neonatal intestine to formula feeding, we developed a new method to examine intestinal microcirculation in real time. To visualize the vasculature and circulating blood cells, we used mice carrying the *Rosa^mT/mG^* transgene, which constitutively expresses membrane Tomato GFP, and membrane GFP upon Cre-mediated recombination (Muzumdar et al., 2007). *Rosa^mT/mG^* mice were crossed with *Tie2-Cre* transgenics, which express the Cre recombinase in endothelial and hematopoietic progenitors and their derivatives, including endothelial cells, platelets and leukocytes (Kisanuki et al., 2001; Koike et al., 2018). The intestinal microcirculation in *Rosa^mT/mG/+^;Tie2-Cre* pups was then visualized under two-photon microscopy. A feeding tube was inserted into pups under general anaesthesia, and the terminal ileum was externalized by laparotomy for analysis (Fig. 3A). Two-photon microscopy allowed us to record live submucosal blood flow 500-1000 µM into the intestinal wall (Fig. 3B,C). We quantified blood flow velocity by measuring platelet movement (Fig. 3C, yellow bar). The fast flow in arterioles can be easily distinguished from slow venous flow, in which leukocytes can be observed rolling on the vascular wall (Fig. 3C; Movies 1-6). This method allowed us to compare blood flow dynamics in response to parenteral formula feeding in the immature versus mature intestinal microvasculature.

Submucosal arterioles, which are the primary regulators of intestinal blood flow (Bohlen, 1987; Watkins and Besner, 2013), were significantly dilated 20 min after gavage feeding in P9 pups (Fig. 3D). Importantly, blood flow increased by 178% over the baseline 30 min after feeding in P9 pups. In contrast, blood flow increased only by 21% in P1, and 45% in P5, pups after feeding (Fig. 3D-F; Movies 1-6).

To visualize the villous vasculature towards the intestinal lumen, the intestine was resected and laid laterally under the microscope (Fig. 3G). The capillary plexus in intestinal villi at P1 and P5 was composed mainly of one capillary vessel loop with a few connecting branches. In contrast, villi capillaries form a more complex network at P9. Generally, a more complex capillary network was apparent in the villi of the jejunum compared to the ileum (Fig. 3H). These results suggest that a less mature intestinal microvasculature at P1 and P5, compared to P9, underlies a poor response to parenteral feeding, resulting in exacerbated hypoxia and NEC.

### Endothelial cells promote vasodilation and concomitantly suppress vasoconstriction in the mature intestinal microvasculature

**Fig. 4.**
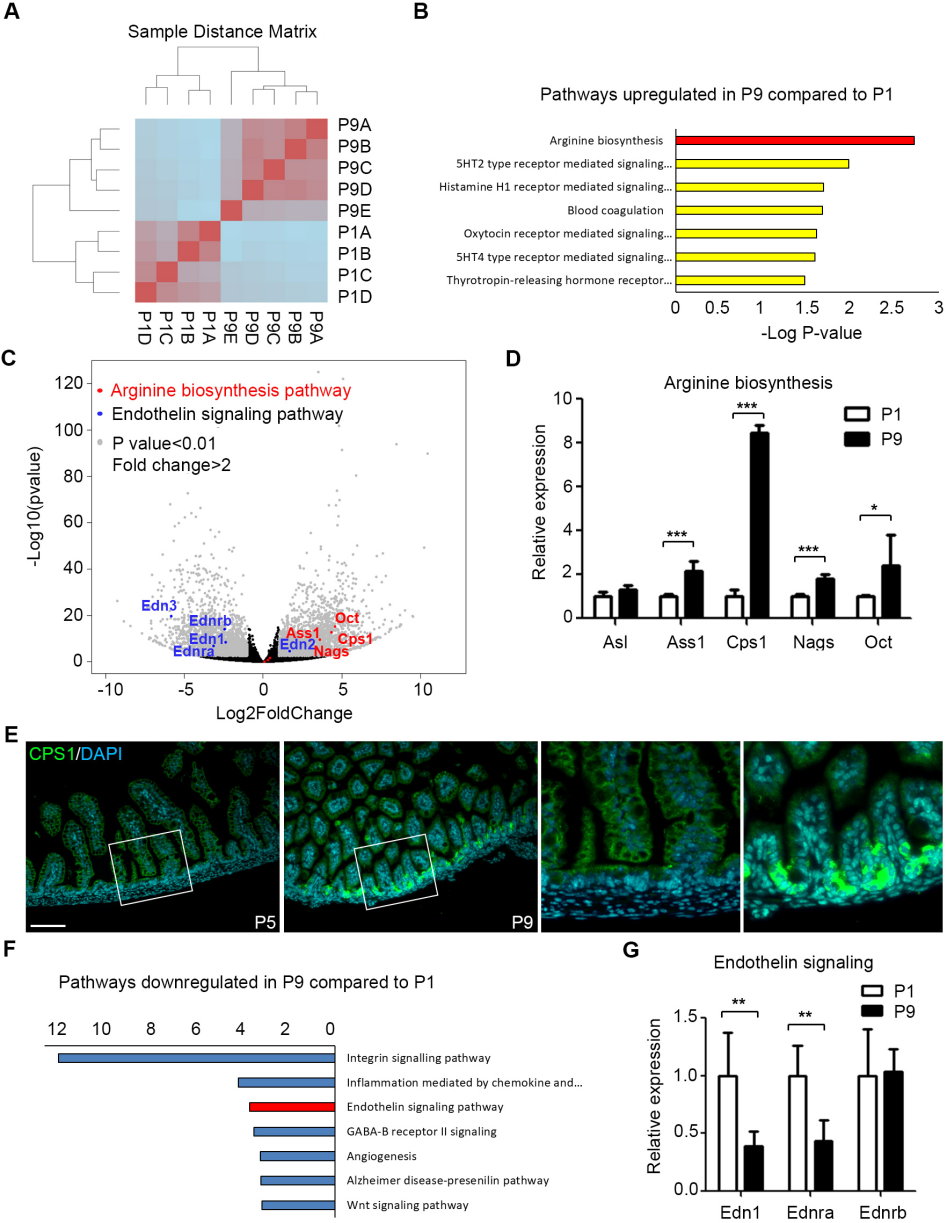
**The mature intestine upregulates arginine synthesis and concomitantly downregulates endothelin (ET-1) signalling pathways.** (A) Hierarchical clustering of the transcriptome of intestinal endothelial cells in P1 (*n*=4) and P9 (*n*=5) *Rosa^mT/mG/+^;Tie2-Cre* pups. (B) Upregulated pathways in P9 compared to P1 intestinal endothelial cells. (C) Volcano plot showing increased expression of genes in the arginine biosynthesis pathway (red), and downregulation of genes in the ET-1 signalling pathway (blue), in P9 versus P1 intestinal endothelial cells. (D) qRT-PCR showing normalized expression of genes in the arginine biosynthesis pathway in ileum from P1 and P9 pups. (E) Immunostaining of CPS1 (green) in P1 and P9 intestine. Nuclei were counterstained with 4′,6-diamidino-2-phenylindole (DAPI). Scale bar: 100 µm. (F) Downregulated pathways in P9 versus P1 pups. (G) qRT-PCR showing normalized expression of genes in the endothelin pathway in ileum from P1 and P9 pups. *P<0.05, **P<0.01, ***P<0.001. All values are mean±s.d.

To uncover endothelial regulators of intestinal microcirculation controlling the response to formula feeding, we compared the global gene expression profile of endothelial cells isolated from the intestine of P1 versus P9 pups by high-throughput sequencing of RNA (RNA-seq). GFP-positive endothelial cells were isolated from *Rosa26^mT/mG/+^;Tie2-Cre* pups by fluorescence-activated cell sorting (Fig. S3A). The purity of endothelial cells was confirmed by quantitative reverse transcription PCR (qRT-PCR), showing high expression of the endothelial marker *Pecam-1* only in GFP-positive cells (Fig. S3B). Hierarchical clustering distinguished unique expression profiles for P1 and P9 endothelial cells (Fig. 4A). Of the ∼25,000 detected transcripts, 4503 genes were differentially expressed (*P*<0.01) by over 2-fold in P9 versus P1 intestinal endothelial cells: 2228 genes were upregulated and 2275 were downregulated. Gene ontologies associated with differentially upregulated genes revealed arginine biosynthesis as the most significantly upregulated pathway in P9 mice (Fig. 4B). Among seven genes involved in arginine biosynthesis, four [carbamoyl phosphate synthase 1 (*Cps1*), argininosuccinate lyase (*Asl*), argininosuccinate synthase 1 (*Ass1*), ornithine transcarbamylase (*Cct*; also known as *Otc*) and N-acetylglutamate synthase (*Nags*)] were significantly upregulated in P9 intestinal endothelial cells (Fig. 4C). qRT-PCR on sorted endothelial cells confirmed increased expression of arginine-biosynthesis-enzyme-encoding genes (Fig. 4D). *Csp1* was the most significantly upregulated gene in P9 intestine endothelial cells. Accordingly, immunofluorescence revealed increased protein at the base of the villi in P9 compared with P1 pups (Fig. 4E). Decreased arginine biosynthesis could reduce NO production and contribute to abnormal microcirculation and NEC in the premature intestine. Our RNA-seq identified endothelin-1 (ET-1) signalling as one of the most downregulated pathways in P9 endothelium (Fig. 4F). ET-1 and Ednra mediate vasoconstriction (Watkins and Besner, 2013). qRT-PCR confirmed decreased expression of the ET-1-encoding gene (*Edn1*) and its receptors [A (*Ednra*) and B (*Ednrb*)] in P9 intestinal endothelial cells (Fig. 4G). These results suggest that promoting vasodilation by upregulating arginine biosynthesis, while concomitantly suppressing vasoconstriction by suppressing ET-1 signalling, allows the P9 intestine to increase blood flow after parenteral formula feeding, thus preventing hypoxia, inflammation, mucosal damage and NEC.

### Promoting vasodilation in the microvasculature in the immature intestine reduces intestinal damage and NEC severity

**Fig. 5.**
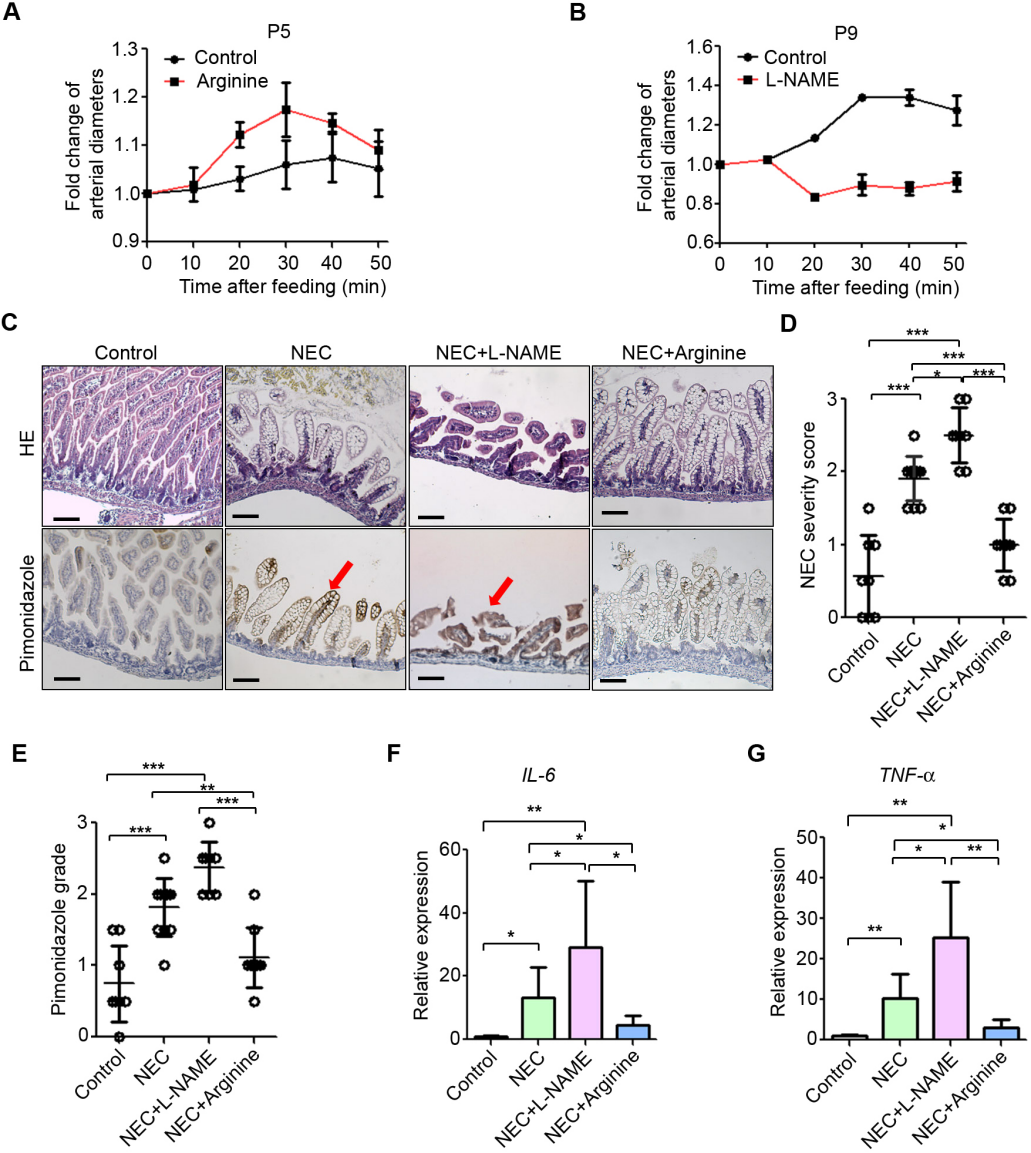
**Inhibition of nitric oxide synthesis compromises postprandial hyperaemia and increases NEC severity, whereas arginine supplementation reduces NEC severity.** (A) Micrographs from two-photon microscopy were used to quantify arterial diameter at several time points after gavage feeding of P5 pups with formula supplemented with arginine. (B) Quantification of arterial diameter change at several time points after gavage feeding of P9 pups with formula supplemented with L-NAME. (C) HE and pimonidazole staining in ileum of control (*n*=8), NEC (*n*=11), NEC+L-NAME (*N*=8) and NEC+arginine pups (*n*=9). Arrows point to damaged villi. (D) NEC severity score. (E) Pimonidazole grading. (F) qRT-PCR of *Il-6* in ileum. (G) qRT-PCR of *Tnf-a* in ileum. Scale bars: 100 μm. *P<0.05, **P<0.01, ***P<0.001. All values are mean±s.d.

Our results suggest that arginine is insufficient in the immature postprandial intestine, raising the possibility that arginine supplementation could increase intestinal blood flow to prevent hypoxia, mucosal damage and development of NEC. To test this possibility, we fed P5 pups with formula supplemented with arginine and measured intestinal microcirculation *in vivo*. Arteriole diameter in the intestine was significantly greater in P5 pups fed with formula supplemented with arginine than in those fed with formula without arginine (Fig. 5A). Decreased intestinal damage and hypoxia, as revealed by decreased pimonidazole staining, and decreased expression of *Il-6* were observed in NEC pups with arginine supplementation compared to other experimental NEC pups (Fig. 5C-F). In contrast, reduced postprandial hyperaemia at P9, as shown by decreased microvascular diameter 20 min after feeding, was observed in pups fed with formula with Nω-nitro-L-arginine methyl ester hydrochloride (L-NAME), a NO synthase inhibitor, compared to control pups (Fig. 5B). Accordingly, NO synthesis inhibition by L-NAME supplementation exacerbated mucosal abnormalities and hypoxia, and induced higher expression of *Il-6* and *Tnf-a* (Fig. 5C-G). Thus, insufficient microvascular dilation and blood flow in the immature intestine in response to feeding underlies mucosal damage in NEC. These results suggest that modulating NO synthesis could be used to prevent the effects of formula feeding and NEC in premature infants.

## DISCUSSION

Our study demonstrates that formula feeding induces mucosal hypoxia and damage in experimental NEC. Feeding-induced gut hypoxia is associated with poor postprandial intestinal hyperaemia in the early life of neonatal pups. Blocking NO-dependent postprandial hyperaemia increases NEC severity, whereas arginine increases postprandial intestinal microcirculation and reduces NEC severity. This study addresses three critical questions regarding the pathogenesis of NEC: (1) What is the basis for intestinal hypoxia/ischaemia in the development of NEC? (2) How does enteral feeding increase the risk of NEC? (3) Why are premature infants more prone to NEC?

Besides inflammation, NEC is characterized by hypoxia/ischaemia (Ballance et al., 1990; Chen et al., 2016b). The basis for hypoxia in NEC is unclear (Uauy et al., 1991; Yu et al., 1984). In this study, we demonstrated that hyperosmolar formula feeding triggers intestinal mucosal hypoxia and NEC in neonatal mice. Our results suggest that intestinal hypoxia is primarily induced by gavage feeding after birth, which is consistent with the later onset of NEC. Feeding increases intestinal oxygen consumption (Ward et al., 2014). This increased need for oxygen is provided by postprandial intestinal hyperaemia (Nowicki et al., 1983). We found that the mouse intestinal microcirculation responds poorly to feeding in early postnatal life. This finding is consistent with induction of intestinal hypoxia by formula feeding in younger mice (Fig. 2). Indeed, premature infants exhibit reduced postprandial splanchnic hyperaemia compared to adults (Havranek et al., 2015; Sieber et al., 1991). In addition, abnormal blood flow in the superior mesenteric artery in fetuses and infants is associated with feeding intolerance and susceptibility to late-onset NEC (Havranek et al., 2015; Murdoch et al., 2006). Therefore, a limited response of the immature intestinal microcirculation to the challenge imposed by formula feeding could predispose to NEC.

Intestinal blood flow is regulated by various neural, humoral, paracrine and metabolic pathways (Matheson et al., 2000). Among those, endothelial-derived vasodilators are crucial for neonatal intestinal microcirculation (Watkins and Besner, 2013). Our whole-genome expression profiling revealed higher expression of *ET-1* (*Edn1*), *Ednra* and *Ednrb* in intestinal endothelium in P1 than in P9 pups. This agrees with previous studies showing that ET-1 activity in the bowel is greater in neonatal piglets than in older pigs (Nankervis and Nowicki, 2000). Interestingly, both NO production and NO-mediated vasodilation are also greater in newborn than older piglets (Nankervis and Nowicki, 1995). Increased NO might counteract the vasoconstrictive effect of ET-1. Although NO production is high in young piglets, the NO precursor, arginine, is remarkably low in premature infants (Contreras et al., 2017). Indeed, our results show that out of seven enzymes in the arginine biosynthesis pathway, four were lower in P1 than P9 intestinal endothelium. The insufficient endogenous arginine synthesis could limit NO production and impair vasodilation in the postprandial intestine. We found that supplementing arginine increases postprandial hyperaemia and prevents experimental NEC. Clinical trials also proved the benefit of arginine supplementation in preventing NEC (Shah et al., 2017). In agreement with a previous report (Yazji et al., 2013), we found that both fresh and pasteurized human breast milk contain higher levels of the NO precursor sodium nitrate than formula (Fig. S4), which might help to explain the protective effects of breast milk in NEC.

Formula feeding is associated with stunted growth (Miyake et al., 2016), and villous atrophy, which is present in our NEC model, is associated with starvation in mice (Song et al., 2009), opening the possibility that undernutrition might contribute to NEC in our model. Our results showing that hypoxia and an inflammatory response are activated upon a single formula feed in DF pups (Fig. 2) suggest that these readouts are independent of nutritional state in our model. Accordingly, formula supplementation with arginine reduced NEC in gavage-fed pups. However, the possibility remains that, in addition to increasing microvascular dynamics in the intestine, arginine supplementation might have had a positive effect on the overall nutrition of the pups. Further research is required to determine the nutritional value of formula feeding in mice, and the contribution of undernutrition in NEC development.

Our finding that insufficient oxygen supply by a deficient response of the immature intestinal microvasculature to feeding contributes to NEC opens new possibilities for early diagnosis. This notion is supported by recent studies showing that near-infrared spectroscopy (NIRS) can detect changes in tissue oxygenation after feeding in an NEC model in piglets (Zamora et al., 2015). In addition, NIRS can distinguish states of NEC complication in preterm infants (Schat et al., 2016).

In summary, our study indicates that limited microvascular dynamics in the premature intestine prevents meeting the increased oxygen demand after feeding. Parenteral feeding in premature infants can push oxygen consumption beyond its supply, resulting in intestinal hypoxia, leading to NEC. This is in agreement with recent trials showing that increasing oxygen saturation to 91-95% from 85-89% in premature infants improved the clinical outcome and decreased mortality (Boost-II Australia and United Kingdom Collaborative Groups et al., 2016). Our findings also suggest that modulating intestinal circulation by targeting ET-1 and NO pathways is a viable strategy to prevent and treat NEC, which continues to be a devastating disease.

## MATERIALS AND METHODS

### Mice

This study was approved by the Animal Care Committee (ACC) at the Hospital for Sick Children (N32238). All experiments were performed following standard operating procedures established by The Centre for Phenogenomics and approved by the institutional ACC. NEC was induced in C57BL/6 mice between P5 and P9 by hyperosmolar formula gavage feeding, oral LPS and exposure to systemic hypoxia, according to an established protocol (Zani et al., 2016). To minimize potential effects of undernutrition in our analysis, pups were fed 50 µl/g three times a day, at 07:00, 14:00 and 22:00. This volume is equivalent to that fed to human premature infants (150 ml/kg/day). The formula used was Esbilac Puppy Milk Replacer Powder (PetAg). To investigate the function of formula feeding on intestinal mucosal damage, we randomly assigned P5 mouse pups to four groups:

1. DF: control pups fed by the dam's breast milk.
2. DF+LPS: pups fed by the dam's breast milk and gavage fed with LPS. Pups were only transiently separated from the mother for sham gavage feeding three times a day without infusion of formula to minimize potential stress of maternal separation and gavage feeding on the intestine (Li et al., 2017).
3. Formula+LPS: pups separated from the mother and gavage fed with formula and LPS. The formula provides 187 kcal/kg/day.
4. Formula+LPS+hypoxia: pups separated from the mother and gavage fed with formula and LPS. Pups were exposed to hypoxia (5% O_2_) before each feeding.

To minimize potential confounding effects of maternal separation in our analysis, we focused on ileal tissue, which is not affected by maternal separation (Zani et al., 2016).

To test the role of arginine and L-NAME in experimental NEC, arginine (240 mg/kg/day, Sigma-Aldrich) or L-NAME (60 mg/kg/day, Sigma-Aldrich) was added to the formula prior to gavage feeding. At P9, 60 mg/kg pimonidazole (Hypoxyprobe, MA, USA) was injected intraperitoneally before the last feeding. Mice were euthanized 90 min after feeding.

For single gavage feeding, pups at P1, P5 and P9 were fasted for 8 h and then injected with pimonidazole and fed with PBS or hyperosmolar formula. *Rosa^mT/mG/+^;Tie2-Cre* mice, which express GFP in endothelial and hematopoietic progenitor cells and derivatives, were generated by crossing *Tie2-Cre* (Proctor et al., 2005) with *Rosa^mT/mG/+^* (Muzumdar et al., 2007) mice.

### Haematoxylin and Eosin staining and immunohistochemistry

Ileal tissues were harvested, formalin-fixed, paraffin-embedded and stained with Haematoxylin and Eosin or subjected to immunohistochemistry for pimonidazole (anti-pimonidazole antibody HP1-100; Hypoxyprobe; 1:100) and CPS-1 (anti-CPS-1 antibody; Abcam, Cambridge, USA; 1:250). Histologic sections were blindly assessed by two independent investigators (Y.K. and H.M.) following a published scoring system for NEC severity and pimonidazole levels (Chen et al., 2016c).

### qRT-PCR

Total RNA was purified using TRIzol Reagent (Invitrogen) and reverse transcribed using qScript cDNA synthesis kit (QuantaBio, Beverly, MA, USA). qRT-PCR was performed using a CFX96 Real-Time System (Bio-Rad). All samples were run in triplicate and gene expression was normalized to the housekeeping genes *RPLO*, *Pgk-1* and *TBP* (also known as *Ppbp*), according to previous publications (Egan et al., 2016; Wang et al., 2010). Primer sequences are listed in Table S1.

### Isolation of intestinal epithelial cells and western blotting

Intestinal epithelial cells were isolated as previously described (Zeineldin and Neufeld, 2012). Briefly, ileum samples were dissected from the mesentery and rinsed in cold PBS. The lumen was flushed with cold PBS and cut open longitudinally. Intestinal segments were incubated in 0.04% sodium hypochlorite on ice for 15 min, transferred to cold solution B (2.7 mM KCl, 150 mM NaCl, 1.2 mM KH_2_PO_4_, 680 mM Na_2_HPO_4_, 0.5 m MDTT and 2.5 mM EDTA) and incubated for 30 min with gentle agitation. Tissue was disaggregated gently by pipetting a few times, and the supernatant containing epithelial cells was collected and centrifuged at 1000 ***g*** for 10 min. Pellets were then lysed in tissue extraction buffer. Western blotting was conducted with 20 µg epithelial lysate. Blots were probed with anti-HIF-1α (SC53546, Santa Cruz Biotechnology; 1:50) and β-actin (4967, Cell Signaling Technology; 1:500) antibodies at 4°C for 72 h and 24 h, respectively (Fig. S2). Quantitative measurement of protein expression was performed using Image Studio Lite (LI-COR Biosciences).

### *In vivo* two-photon microscopy of intestinal microcirculation

A 1.9F feeding catheter was inserted into *Rosa^mT/mG/+^;Tie2-Cre* pups anesthetized with isoflurane (Fig. 3A). Pups were secured on a warm plate under a two-photon microscope. The terminal ileum (3-4 cm proximal to the cecum) was externalized by laparotomy and fixed loosely between a holder loop and a coverslip placed slightly above the abdominal wall. Submucosal haemodynamic was recorded every 10 min from 0 to 50 min after feeding. Vessel diameter was measured on at least six points, usually just above the bifurcation on arterioles, before and after feeding. Arterial blood flow velocity was measured using flow image correlation spectroscopy, as previously described (Sironi et al., 2014). Arterial blood flow was calculated using the following formula: π(diameter/2)^2^ × velocity. White blood cells are larger and move more slowly than the smaller platelets. These properties allowed identification and analysis of their movement in live microcirculation videos (Koike et al., 2018). After recording the time course of the haemodynamic response to feeding, small pieces of jejunum and ileum were resected and cut open for observation of the villus microvasculature under a two-photon microscope.

### Endothelial cell sorting and RNA-seq

P1 and P9 *Rosa^mT/mG/+^;Tie2-Cre* pups were fasted for 8 h and fed with PBS or hyperosmolar formula. The small intestine was harvested 90 min after feeding. After rinsing and flushing with cold PBS three times to remove blood, the lumen was opened longitudinally and cut into small segments. Intestinal cells were dissociated from tissue by incubating in TrypLE™ Express (Thermo Fisher Scientific) for 30 min at 37°C. Cells were then washed and sorted for GFP-positive and -negative cells in a flow cytometer (MoFlo XDP cell sorter). RNA was isolated using a TRIzol^®^ Plus RNA Purification Kit (Zymo Research), and RNA integrity was verified using an Agilent Bioanalyzer (Agilent Technologies). cDNA synthesis was performed with a SMART-Seq Ultra low-input RNA kit (Clontech Laboratories). A cDNA library was then prepared used a Nextera XT Library Preparation Kit (Illumina) and sequenced on an Illumina HiSeq 2500 platform. Sequencing reads were trimmed using Trimmomatic then mapped to the mouse genome (mm10) using STAR (Dobin et al., 2013). Mapped read counts were obtained using featureCounts (Liao et al., 2014). Differential expression analysis was performed using DESeq2 (Love et al., 2014). Clustering of genes involved in arginine synthesis and the endothelin pathway was performed using Cluster 3.0 and visualized with Java Treeview. Enrichment of pathways in differentially expressed genes was determined using PANTHER (Mi et al., 2017).

### Breast milk

Surplus human breast milk from four healthy donors was collected at the Hospital for Sick Children's nutritional services (Toronto, ON, Canada) and stored and processed as described previously (Wu et al., 2019).

### Statistical analysis

Data were tested for normality of distribution (Kolmogorov–Smirnov test) and compared using parametric or nonparametric tests as appropriate. Statistical analysis was performed using two-tailed Student's *t*-test or one-way ANOVA with Bonferroni correction. Data are presented as mean±s.d. *P*<0.05 was considered significant.

## Supplementary Material

Supplementary information
