# Supplementary material for: Formula feeding and immature gut microcirculation promote intestinal hypoxia, leading to necrotizing enterocolitis
Source: Dis Model Mech. 2019 Dec 9;12(12):dmm040998. doi: 10.1242/dmm.040998 (PMC6918740; doi:10.1242/dmm.040998)
Supplement: Supplementary information [file dmm-12-040998-s1.pdf]

Table S1. Primer sequences for RT-PCR.

Gene	Forward primer	Reverse primer
ASL	CCGGCATCTGTGGAATGTG	GTTGCGACTTCGTCCTGTGT
ASS1	CTCCTGCATCCTCGTGTGG	GCTCACATCCTCAATGAACACC
CPS1	ACATGGTGACCAAGATTCCTCG	TTCCTCAAAGGTGCGACCAAT
EDN1	TTTCCCGTGATCTTCTCTCTGC	CTGAGTTCGGCTCCCAAGAC
EDNRA	GCAGCCACATGGAAGACTTC	CATTGAGCCATTGCTAGGCA
EDNRB	AAGCCACGCTGTCACTTCTC	GAGGAACGCATCAGACTGGA
GLUT-1	AGAGAGACCAAAGCGTGGTG	GCAGTTCGGCTATAACACTGG
GFP	ACTTCAAGATCCGCCACAAC	GTGTCAGGTAGTGGTTGTCG
HIF-1α	AAGTGGCAACTGATGAGCAA	GGCGAGAACGAGAAGAAAAA
IL-6	CCAATTTCCAATGCTCTCCT	ACCACAGTGAGGAATGTCCA
NAGS	CTCTAGCCAAGGCTTTGCAG	GATCTTCTGACTGTTATTCCGCA
OCT	AGGGTCACACTTCTGTGGTTC	CAGAGAGCCATAGCATGTACTG
PECAM-1	GTACAGCCTCCAACAGAGC	TGACCACTCCAATGACAACC
PGK-1	GATGAGGGTGGACTTCAACG	AGGTGGCTCATAAGGACAAC
RPLO	GGCGACCTGGAAGTCCAACT	CCATCAGCACCACAGCCTTC
TBP	CAAACCCAGAATTGTTCTCCTT	ATGTGGTCTTCCTGAATCCCT

Disease Models & Mechanisms • Supplementary information

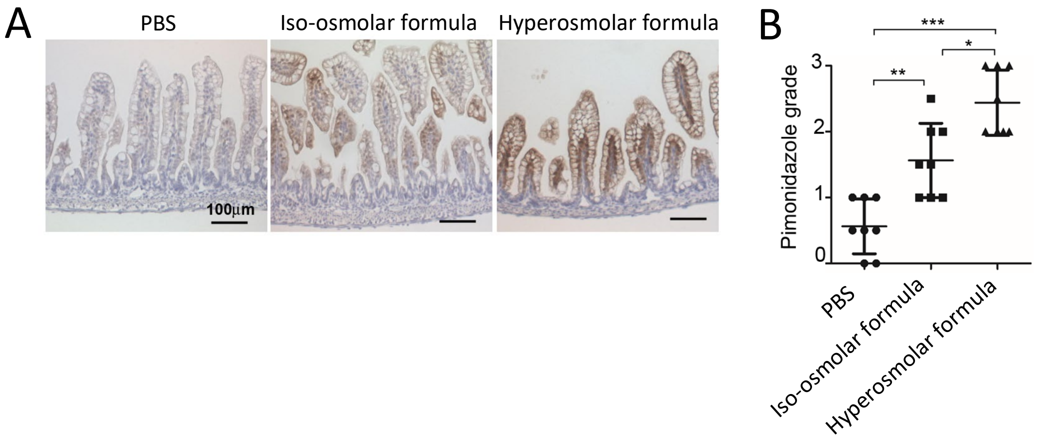

Fig. S1. Iso-osmotic milk induced intestinal hypoxia is less severe than that of hyperosmolar milk. (A) Pimonidazole staining (left) and, (B) Pimonidazole grade of ileal tissue from P5 pups fed with PBS, iso-osmolar formula (1:1 dilution of hyperosmolar formula) and hyperosmolar formula (n=8 per group). Scale bar =100µM. Error bars represent the mean ± s.e.m. **P*<0.05, ***P*<0.01, ****P*<0.001.

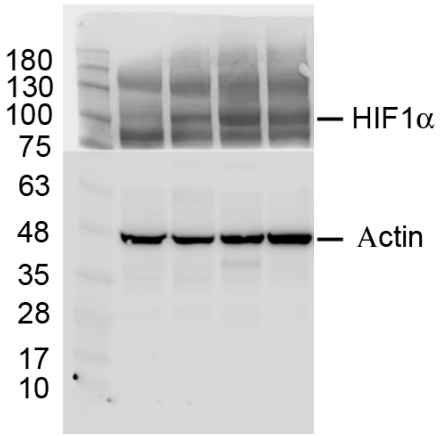

Fig. S2. Uncut membrane of western blot

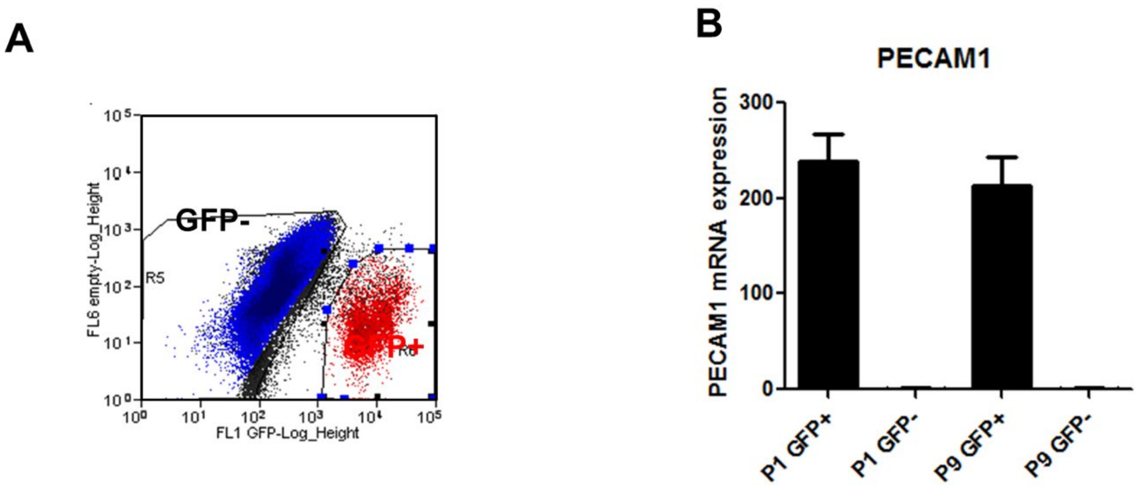

Fig. S3. Isolation of intestinal endothelial cells for RNAseq. (A) representative FACS plot for GFP+ (red) and GFP- (Blue) intestinal endothelial cells from *Rosa*$^{mT/mG/+}$*;Tie2-Cre* pups. (B) qRT-PCR showing enrichment of the endothelial marker *PECAM-1* in GFP+ cells.

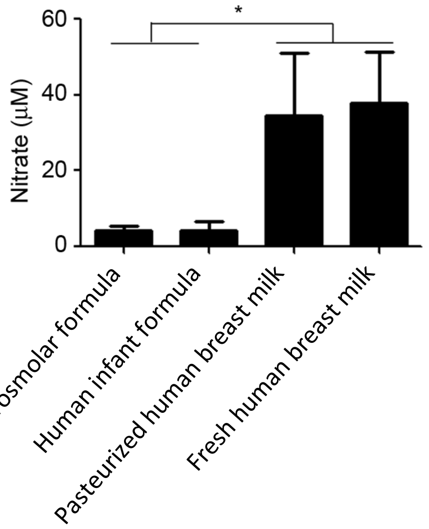

Fig. S4. Nitrate concertation in formula and human breast milk. Nitrate was measured in triplicate on deproteinized puppy formula, infant formula (Similac, Abbott Nutrition, OH), pasteurized donor breast milk and fresh breast milk. (* P<0.05)

Disease Models & Mechanisms • Supplementary information

Supplementary movies

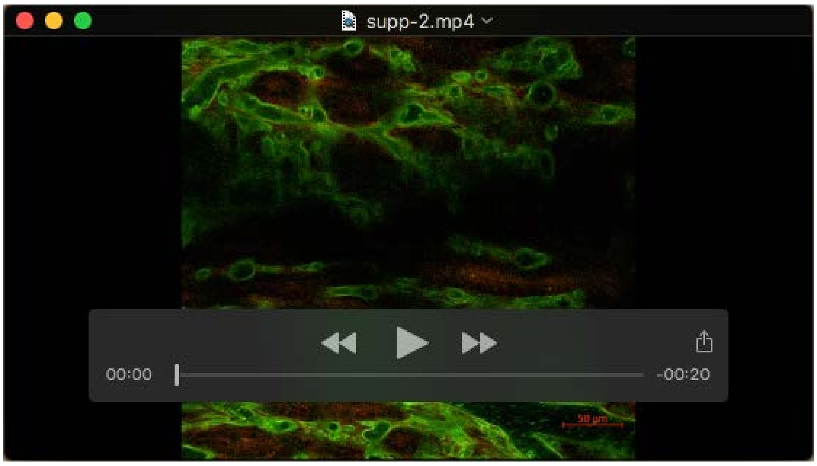

Movie 1

Two-photon microscopy showing intestinal submucosal microcirculation in P1 before feeding.

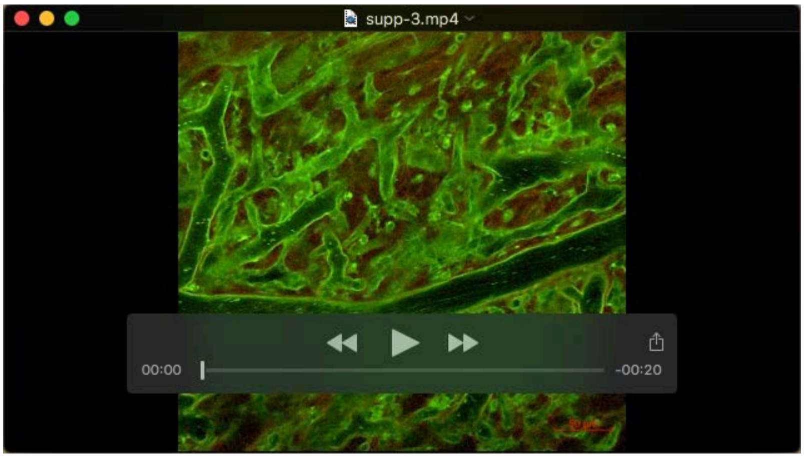

Movie 2

Two-photon microscopy showing intestinal submucosal microcirculation in P5 before feeding.

Disease Models & Mechanisms • Supplementary information

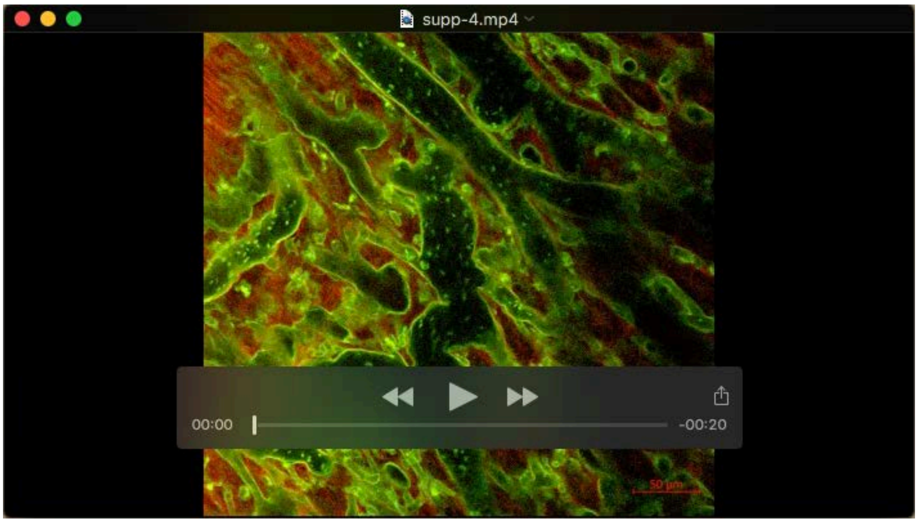

Movie 3

Two-photon microscopy showing intestinal submucosal microcirculation in P9 before feeding.

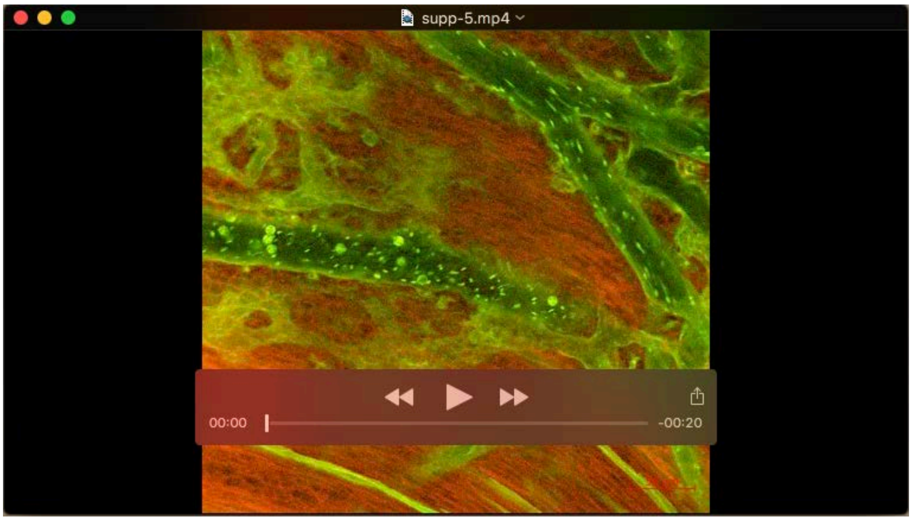

Movie 4

Two-photon microscopy showing intestinal submucosal microcirculation in P1 after feeding.

Disease Models & Mechanisms • Supplementary information

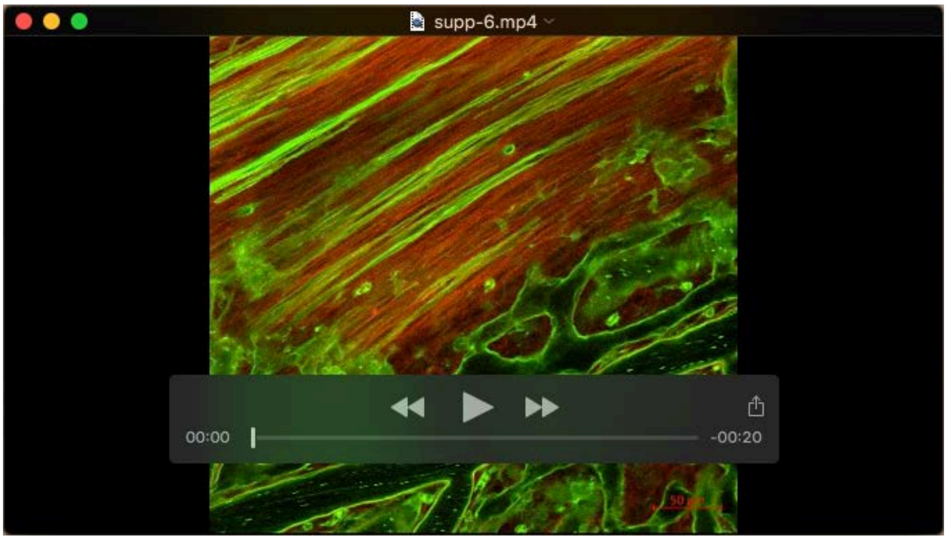

Movie 5

Two-photon microscopy showing intestinal submucosal microcirculation in P5 after feeding.

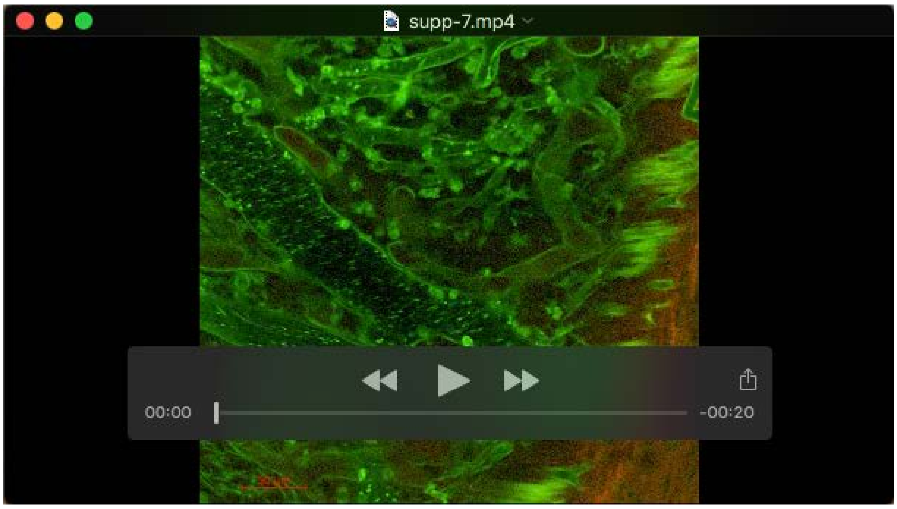

Movie 6

Two-photon microscopy showing intestinal submucosal microcirculation in P9 after feeding.
